# 

**Published:** 1887-08-27

**Authors:** 


### THE HOSPITAL

An Institution, Family, and Parochial Journal of Hospitals, Asylums, & all Agencies for the Care of the Sick, Criticism & News.

### CONTENTS.

**Tinker or Prophet?** - 357
**Words of Consolation.**—XLVII. The Sufferings of Christ - 358
**The Doctor's Pulpit.**—Blood-making - 359
**Dispensaries for Sailors.** By PIETRO MICHELLI - 360
**The Armour Skeleton.** By W. E. FOTHERGILL - 361
**Religio Medici** - 363
**Notes and News.**—Donations in Aid of the Proposed Cottage Hospital at Wellingborough.—The Study of Deep-sea Animals.—Jubilee Memorial Cottage Hospital at Falkirk.—The Queen and the Women's Jubilee Offering.—The Launceston General Hospital, Tasmania.—Vacancies.—Scarlet Fever in London.—Munificent Donation to the Montrose Infirmary.—Decorations for Nursing Sisters.—The Edinburgh Royal Hospital for Sick Children.—Lady Brooke and the Southend Hospital.—The Bournemouth Hospital Scheme.—Meeting of the Hospital Saturday Fund Delegates.—The Hospitals Association Reference Library.—West Bromwich Hospital, etc. - 364
**Annotations.**—Registered Plumbers.—Sea Water for Inland Towns.—Swimming Baths - 367
**Kate Mountstephen.** By C. G. FURLEY.—Chapter I.—The Test of Time - 368
**The National Pension Fund** - 370
**Farinaceous Foods** - 371
**A French Hospital: An Inside View.** By an ENGLISHMAN AND FORMER PATIENT - 371
**Incidents of Animal Life.** By the Rev. F. O. MORRIS, B.A. About Birds - 372
**The Children's Corner.**—Puzzles, Answers, etc. - vi
**Amusements with Profit** - vi

